# Atomic scale interface design and characterisation

**DOI:** 10.3762/bjnano.6.174

**Published:** 2015-08-10

**Authors:** Carla Bittencourt, Chris Ewels, Arkady V Krasheninnikov

**Affiliations:** 1 Chimie des Interactions Plasma-Surface (ChIPS), CIRMAP, Research Institute for Materials Science and Engineering, University of Mons, 7000 Mons, Belgium; 2 Institute des Matériaux Jean Rouxel, IMN, Université de Nantes, CNRS UMR6502 Nantes, France; 3 Department of Applied Physics, Aalto University, Finland; 4 Institute of Ion Beam Physics and Materials Research, Helmholtz Zentrum Dresden-Rossendorf, Germany

**Keywords:** carbon, first-principles simulations, interface, nanomaterials, nanoscale, oxides, spectromicroscopy

While many major strides have been made in the last three decades concerning the synthesis, characterisation and properties of individual nanoobjects, their technological uptake necessarily requires integration into devices. To achieve this, detailed characterization, design and control of the interface between nanoobjects and their environment is essential. Nanomaterials differ significantly from their bulk counterparts and one important aspect of this is their high surface to volume ratios. For this reason interface properties typically dominate the behavior of nanomaterials. As a consequence, one immediately faces the problem of designing and controlling surfaces and, in case of embedded or layered systems, interfaces on atomic scales.

Engineering of electrical contacts is required for nanostructure integration. The performance of electronic devices based on nanostructures is affected by a potential barrier at the metal–nanostructure contact, thus investigation of the metal–nanostructure interaction aiming at understanding the physics and chemistry of this interface is critical for the design of optimal devices [1–3]. Potential applications of nanostructures such as spintronics, or light absorption and emission devices require doping [4–5]. In this context, conventional bulk doping techniques must be adapted, given the large surface to volume ratio of nanostructures, surface segregation of dopant atoms is a severe drawback. Therefore detailed knowledge and control of the physical and chemical properties and processes occurring at the surface on a nanoscale are of crucial concern.

Nanostructured materials show a great application potential in the areas of nanoelectronics, catalysis, and light harvesting/energy storage, an excellent example being the capability of titanate nanoribbons to simultaneously harvest and store energy in photobatteries [6]. In such systems, the exact nature of the interface between different nanostructures is the decisive factor determining the solar harvesting efficiency. The optimal integration of nanostructured materials into larger scale devices and their diverse applications, both are often hindered by technological constraints arising from the lack of precise knowledge and control of the interface formation at the nanoscale. To date, the main obstacle for the scientific community was posed by the limits of some conventional characterization tools when applied at the nanoscale: Tools such as vibrational and electronic spectroscopy were typically macroscopic probes sampling areas in the range of square millimeters or square micrometers, at best. Hence, these techniques are inadequate for the characterization of nanoscale interfaces. The development of new experimental tools and the detailed understanding of their impact on the sample structure will therefore provide a substantial contribution to the design and optimization of novel devices [7].

Addressing design, control and characterization of nanoscale interfaces and the development of new tools to facilitate this is a vast area of research, requiring multidisciplinary collaboration between chemists, physicists, biologists and engineers across a wide range of disciplines. It is clearly a very broad topic for a single Thematic Series, and for this reason the current volume has been jointly edited by Carla Bittencourt, Chris Ewels and Arkady Krasheninnikov, with the reviews covering key areas of advanced synthesis methods, controlled functionalization and post-processing routes, developments in nanoscale characterization tools as well as theoretical modeling of structure and dynamics, and electronic properties. Finally some reviews cover key examples of successful nanomaterial integration.

Recent drives to improve and refine nanomaterial synthesis routes have adopted different approaches. The increasingly important role played by organic chemists in the field of nanocarbon design has led to new families of atomically designed materials such as cycloparaphenylenes, the ultimate “short nanotubes” [8]. At slightly larger scales, atomic layer deposition allows the design of atomic-scale hybrid materials such as TiO_2_ deposition on carbon nanotubes [9]. Post-growth engineering of nanomaterials is also considerably refined, for example new understandings of diffusion processes during growth and oxidation allow for the engineering of hollow nanostructures using the Kirkendall effect [10], the filling of carbon nanotubes and nanofibers to tune their properties [11], or the use of electron irradiation to produce carbon atom chains [12].

Graphene and related materials such as nanotubes, polycrystalline nanoparticles [13] and carbon onions [14] have seen significant progress in preparing samples of reasonable size and quality [15] and form a worldwide actively pursued field of research on understanding and modifying their properties [16–17]. Besides dealing with fundamental questions, contributions emphasize possible applications of the remarkable physical and chemical properties of this class of materials such as thermoelectrics [18], plasmonics [19] and gas sensing [20]. In the future, spatial control of doping may allow for surface modifications in a laterally controlled way. Controlled functionalization will allow for optimal integration of nanostructures in electronic devices but also for their use in biological applications. It is important to mention the use of carbon nanomaterials in neuroengineering, where these nanomaterials have been used as substrates for neural growth, as drug delivery systems and as electrodes for both extra cellular recordings and for in vivo recordings [21]. Finally, although carbon is often seen as the “poster child” of nanomaterials, it is important not to forget the expanding range of applications for related nanomaterials such as boron nitride nanotubes [22] and silicon and germanium nanocrystals [23].

Recent developments in X-rays collimation optics triggered the interest in synchrotron radiation-based techniques for the study of nanostructures. In this context, X-ray microscopy has shown to be a powerful tool for chemical analysis of radiation-sensitive nanomaterial [7]. Combining the chemical and magnetic information provided by XPEEM with the structural sensitivity of LEEM has created a complete characterization tool of material properties at the nanometer length scale. This can be complimentary with conventional XPS, whose peak assignments for identifying heteroatom doping behavior in nanocarbons are reviewed by Susi et al. [24].

Another important characterization tool for nanomaterials has emerged from the introduction of aberration correctors in transmission electron microscopes (TEM), which brings both increased spatial resolution and energy resolution allowing imaging at low voltage, crucial for nanomaterials which are sensitive to electron irradiation. Currently, the atomic structure of different nanomaterials can be revealed with a resolution of better than 0.1 nm, in addition to elemental analysis [25].

The analysis of experimental results can significantly profit from the comparison of the images to the results of first-principles calculations. Similarly, for the precise interpretation of experimental scanning tunneling microscopy (STM) and atomic force microscopy (AFM) images, the microscopy knowledge about the local electronic structure of the system is extremely important. Full understanding of the electronic structure and properties of a large number of solids can be credited to first principles simulations, and more specifically density-functional theory (DFT) approaches. In addition, using DFT-based molecular dynamics, the manipulation of nanostructures by SPM tools and the changes made to the system by the characterization tools, e.g., the production of defects under electron irradiation and their evolution over time, can be simulated.

An impressive example of how STM experiments and DFT calculations together can unravel the atomic structure of the material is given in the article by J. A. Lawlor and M. S. Ferreira [26] focused on the identification of dopant impurities in graphene. Synergy effects of TEM and DFT are illustrated in the article by M. Quintana et al. [15], by the examples of imaging molecules and clusters on graphene. Another example is the article by Barmparis et al. on Wulff construction [27], based on first-principle calculations. This method has become quite popular as a tool of choice for the simulation of nanoparticle shapes. In the past decade, many TEM and catalysis experiments were simulated using this multi-scale approach with remarkable success.

First-principle calculations have also provided insight into the electronic properties of low-dimensional materials, for example graphene doping by surface adsorbates [28], and nanosystems structured from misfit layer compounds [29]. Planar and bended misfit structures, such as tubes, scrolls or nanoparticles, with intriguing electronic and magnetic characteristics have been synthesized, while calculations explained their electronic structure and properties.

An important remark is related to the background of all contributions of the present volume. They represent a cross-section of research subjects from members of the European COST Action NMP 0901 “NanoTP”, which ran from 2009 to 2014. The project brought together chemists, physicists, biologists and engineers across a wide range of disciplines, to share their common interest and pool their skills in the field of atomic-scale interface design. At its end the project had nearly 300 members drawn from 26 countries COST countries, along with members in Russia, Australia, Armenia, Belarus, Mexico and Canada. The network allowed researchers to mutually visit laboratories, organise training schools and workshops on diverse subjects including new spectroscopy and microscopy tools, latest developments in atomic scale simulations, and nanocarbon design and application. It helped to launch the ongoing “GraphITA” graphene conference series [30], and produced videos on YouTube explaining graphene and related science for the general public [31–32].

Within its broad scientific and technical objective, secondary objectives were identified including synthesis of novel nanostructures, engineering of metal-nanostructures contacts, development of new tools for characterization and manipulation nanostructures, atomic-scale quantum chemical modelling, and integration in potential devices. All of these are reflected in the 26 reviews presented here. They appropriately demonstrate the richness and diversity within the NanoTP Action, as well the active participation and enthusiasm of the project members which made the project such a success. This collection of articles is a fitting conclusion to the project, and we hope the collection will also serve to stimulate amongst its readers the most important motivation of all: scientific curiosity.

Last but not least, we would like to thank the Production Team of the Beilstein-Institut for their enthusiasm, encouragement, patience and engaged editorial support.

Carla Bittencourt, Chris Ewels and Arkady V. Krasheninnikov

Mons, Nantes and Helsinki, June 2015
